# The Physician’s Visiting List for 1883

**Published:** 1882-12

**Authors:** 


### The Physician's Visiting List for 1883.

This valuable little work has just made its thirty-second annual visit. This work has been so perfected in its various parts and arrangements that it would seem that nothing further is desired, or could be attained. For completeness, compactness and simplicity of arrangement it may fairly be said to stand without a rival for the physician's purposes.

It contains a new table of poisons and their antidotes.

The Metric, or French decimal system of weights and measures and posological tables, showing the relation of our present system of Apothecaries weights and measures to that of the Metric system, giving the doses in both. These, with other interesting matters included, make the work one of value, not only to the general practitioner of medicine, but to those who are engaged in any of the medical specialties. It is neatly gotten up, bound in leather, with tuck. Indeed, to give an idea as to what it is in this respect, it is quite sufficient to say it is the work of P. Blakiston, Son & Co., Philadelphia. For sale by Robt. Clarke & Co. Price, $1. to $3., according to size.

